# Fatal Cytokine Collision: HLH–AIHA in Advanced AIDS—Case Report and Literature Review

**DOI:** 10.3390/reports8030137

**Published:** 2025-08-04

**Authors:** Xiaoyi Zhang, Maria Felix Torres Nolasco, Wing Fai Li, Toru Yoshino, Manasa Anipindi

**Affiliations:** Department of Internal Medicine, Jacobi Medical Center, Albert Einstein College of Medicine, Bronx, New York, NY 10461, USA; torresnm@nychhc.org (M.F.T.N.); liw4@nychhc.org (W.F.L.); yoshirot@nychhc.org (T.Y.); anipindm@nychhc.org (M.A.)

**Keywords:** hemophagocytic lymphohistiocytosis, autoimmune hemolytic anemia, advanced AIDS, cytokine storm, epstein-barr virus, soluble interleukin-2 receptor, hyperferritinemia, etoposide

## Abstract

**Background and Clinical Significance**: Hemophagocytic lymphohistiocytosis (HLH) and autoimmune hemolytic anemia (AIHA) are both life-threatening hematologic syndromes that rarely present together outside of malignancy. Advanced acquired immunodeficiency syndrome (AIDS) creates a milieu of profound immune dysregulation and hyperinflammation, predisposing patients to atypical overlaps of these disorders. **Case Presentation**: A 30-year-old woman with poorly controlled AIDS presented with three weeks of jaundice, fever, and fatigue. Initial labs revealed pancytopenia, hyperbilirubinemia, and elevated ferritin level. Direct anti-globulin testing confirmed warm AIHA (IgG^+^/C3d^+^) with transient cold agglutinins. Despite intravenous immunoglobulin (IVIG), rituximab, and transfusions, she developed hepatosplenomegaly, extreme hyperferritinemia, and sIL-2R > 10,000 pg/mL, meeting HLH-2004 criteria. Bone marrow biopsy excluded malignancy; further work-up revealed Epstein–Barr virus (EBV) viremia and cytomegalovirus (CMV) reactivation. Dexamethasone plus reduced-dose etoposide transiently reduced soluble interleukin-2 receptor (sIL-2R) but precipitated profound pancytopenia, Acute respiratory distress syndrome (ARDS) from CMV/parainfluenza pneumonia, bilateral deep vein thrombosis (DVT), and an ST-elevation myocardial infarction (STEMI). She ultimately died of hemorrhagic shock after anticoagulation despite maximal supportive measures. **Conclusions**: This case underscores the diagnostic challenges of HLH-AIHA overlap in AIDS, where cytopenias and hyperferritinemia mask the underlying cytokine storm. Pathogenesis likely involved IL-6/IFN-γ overproduction, impaired cytotoxic T-cell function, and molecular mimicry. While etoposide remains a cornerstone of HLH therapy, its myelotoxicity proved catastrophic in this immunocompromised host, highlighting the urgent need for cytokine-targeted agents to mitigate treatment-related mortality.

## 1. Introduction and Clinical Significance

HLH is a life-threatening hyperinflammatory syndrome characterized by uncontrolled immune activation, often triggered by infections or malignancies. AIHA, conversely, arises from antibody-mediated erythrocyte destruction and is relatively rarely associated with HLH outside lymphoma contexts [1]. A French nationwide study of 33,403 HIV-infected patients identified 49 cases of AIHA as comorbidity [2]. Another nationwide population study from Taiwan recruiting 19,052 patients with HIV had 23 cases of AIHA. Neither study reported presence of HLH [3]. To date, only two cases of HLH-AIHA overlap have been reported, both in diffuse large B-cell lymphoma (DLBCL) patients [4]. The coexistence of HLH and AIHA in advanced AIDS without malignancy has not been extensively reported in the literature. This gap reflects both the rarity of such overlap and the diagnostic challenges posed by overlapping cytopenias, hyperferritinemia, and organomegaly [5,6]. The interplay between cytokine storms (e.g., IL-2R, IFN-γ) and autoimmune phenomena remains poorly understood.

Inducing components of HLH can be categorized into impaired cytotoxic function [7], underlying inflammatory disorders (lymphoma, leukemia, autoimmune disease), immunosuppression (HIV infection, immunosuppressing medications), and infectious triggers. Viral infection, including EBV, CMV, herpes simplex virus, and, less frequently, RNA virus such as influenza virus or HIV, are recognized as common triggers of HLH [8]. Specifically, EBV and CMV establish life-long infections that chronically stimulate pattern recognition receptors, such as Toll-like receptor, thereby increasing the burden on innate immunity [8,9]. Various mechanisms have been suggested, such as cytokine and chemokine imbalance, inhibition of apoptosis, bone marrow suppression, and natural killer (NK) cell and cytotoxic T cell suppression [8]. Especially in the profoundly immunosuppressed host, even minor inflammatory cues may tip the balance toward a self-perpetuating cytokine loop dominated by interleukin-6, interferon-γ, and other pro-inflammatory mediators. Epstein-Barr virus (EBV) and cytomegalovirus (CMV) co-reactivation is increasingly recognized as a critical trigger for this dual pathology. EBV, through latent membrane protein (LMP1) signaling, induces IL-6 and IFN-γ overproduction, which simultaneously activates macrophages and promotes autoreactive antibody formation [10,11]. CMV exacerbates this process by amplifying IFN-γ release and impairing cytotoxic T-cell function, creating a synergistic cytokine storm that destabilizes immune homeostasis [8,12]. In HIV/AIDS, severe CD4+ T-cell depletion (<50 cells/μL) further cripples EBV-specific immunity, enabling uncontrolled viral replication and molecular mimicry between EBV nuclear antigens (e.g., EBNA-1) and erythrocyte surface proteins (e.g., Band 3) [10,13,14,15]. This mechanism, previously observed in autoimmune disorders like systemic lupus erythematosus, is amplified in AIDS due to profound immunodeficiency, positioning EBV as a dual driver of hyperinflammation and autoimmunity [10,11].

Altogether, these overlapping factors underscore that hyperinflammation in HIV-associated HLH-AIHA is distinctly multifactorial.

We herein present a case of HLH with mixed warm/cold AIHA in an AIDS patient without malignancy.

## 2. Materials and Methods

Clinical information was extracted retrospectively from the electronic medical record of Jacobi Medical Center (JMC), a College of American Pathologists (CAP)–accredited institution that undergoes biennial peer inspection and continuous proficiency testing to ISO 17043 standards (College of American Pathologists, College of American Pathologists).

### 2.1. Laboratory Investigations

All first-line hematology tests—including complete blood count (CBC), differential, and reticulocyte enumeration, etc.—were analyzed on a Sysmex XN-1000 platform that uses fluorescent flow cytometry to generate a comprehensive CBC profile and validated adult reference intervals (sysmex.com, PMC). Routine chemistry (e.g., ferritin, bilirubin, triglycerides, lactate dehydrogenase [LDH]) and immunoassays were processed on the Abbott ARCHITECT i2000SR, a chemiluminescent microparticle system with a throughput of 200 tests h^−1^ (corelaboratory.abbott, PMC). Assay performance was verified using daily two-level internal controls and enrolment in CAP proficiency programs.

Specialized tests unsuitable for in-house processing were forwarded within one hour of phlebotomy to Northwell Health Laboratories. Serum soluble IL-2 receptor-α (sIL-2R/CD25) was quantified with the Quantikine high-sensitivity Enzyme-linked immunosorbent assay (ELISA) (R&D Systems), whose validated adult reference range is 175–858 pg mL^−1^. EBV and CMV DNA loads were measured on the dual-target cobas 6800/8800 real-time polymerase chain reaction (PCR) system; the analytical sensitivity for EBV is 18.8 IU mL^−1^, and the linear range extends to 1 × 10^8^ IU mL^−1^ (Diagnostics, Oxford Academic). Diagnostic thresholds for hyperferritinemia, hypertriglyceridemia, hypofibrinogenemia, or elevated sIL-2R followed the HLH-2004 criteria (NCBI, PMC). All laboratory values were time-stamped and automatically uploaded to the electronic chart, then cross-checked against nursing flowsheets for accuracy.

### 2.2. Literature Review Strategy

Because the objective was illustrative rather than exhaustive, we performed a focused narrative review instead of a full systematic review, consistent with the CARE case-report recommendations for transparency without PRISMA compliance (CARE Case Report Guidelines, DistillerSR). Two authors (XZ, MFN) searched PubMed, Embase, Web of Science, and CNKI for studies indexed from database inception to 1 July 2025 using the following Boolean string: (“hemophagocytic lymphohistiocytosis” OR HLH) AND (“autoimmune hemolytic anemia” OR AIHA) AND (HIV OR AIDS). No language or publication-type filters were applied. After title–abstract screening, full texts were reviewed; we included adult cases with confirmed HLH and AIHA where malignancy was excluded, and we hand-searched reference lists for additional reports. Data items (trigger organism, treatment, and outcome) were abstracted into a predefined spreadsheet; because heterogeneity precluded quantitative synthesis, findings were summarized descriptively. Disagreements were resolved by author consensus without third-party adjudication, a process acceptable for narrative syntheses.

## 3. Case Presentation

A 30-year-old woman with advanced AIDS (CD4 14 cells/μL, non-adherent to antiretroviral therapy) presented with three weeks of jaundice, fatigue, and fever. Her functional status was independent but fatigued (Eastern Cooperative Oncology Group [ECOG] 1–2). Initial evaluation (18 October 2024) revealed pancytopenia (hemoglobin 9.2 g/dL, platelets 141 K/μL, absolute neutrophil count 3.01 K/μL), hyperbilirubinemia (total bilirubin 3.57 mg/dL), and elevated ferritin (6173 (ng/mL). Initial viral serology (13 October 2024) showed evidence of past EBV infection (viral capsid antigen (VCA) IgG > 750 U/mL, early antigen (EA) IgG 45.8 U/mL) with negative Epstein–Barr nuclear antigen (EBNA) IgG, and past CMV infection (CMV IgG 2.24 U/mL) with undetectable viral loads for both viruses. Direct antiglobulin testing confirmed warm autoimmune hemolytic anemia (AIHA; IgG+/C3d+), while transient cold agglutinins (1:64) suggested mixed autoimmune pathology. Despite transfusions, IVIG (1 g/kg × 5 doses), and rituximab (375 mg/m^2^ weekly × 4), her condition rapidly deteriorated over the subsequent weeks.

Sequential viral reactivations emerged as her immunosuppression deepened. By November 13, EBV viremia became detectable (3310 IU/mL) alongside low-level CMV reactivation (<34.5 IU/mL) with positive CMV IgM (44.3 AU/mL). On 23 November 2024, serology confirmed acute EBV reactivation with positive VCA IgM (>160 U/mL) and conversion of EBNA IgG to positive (>600 U/mL), suggesting a complex pattern of viral reactivation rather than primary infection (Table 1).

**Figure 1:**
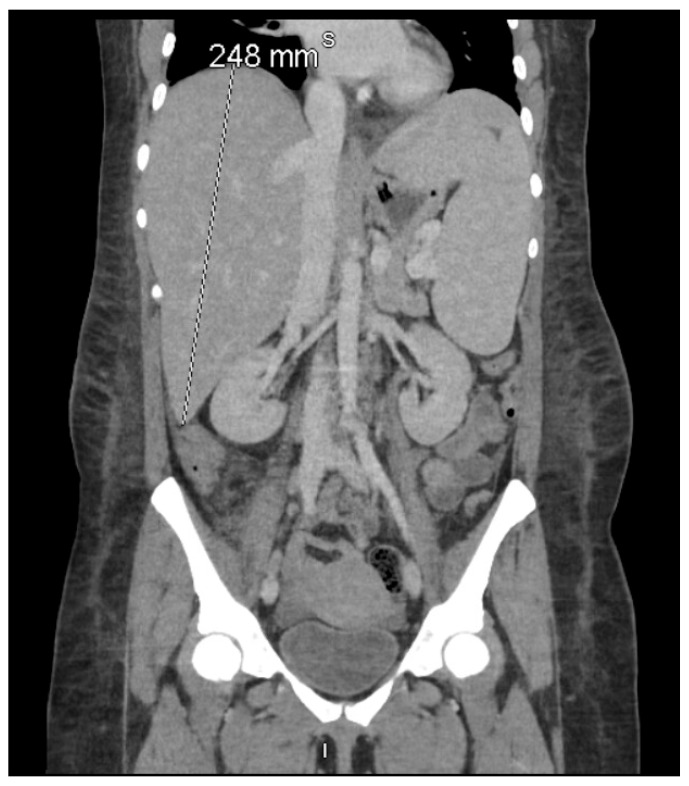
Coronal contrast-enhanced computed tomography (CT) of the abdomen and pelvis demonstrating pronounced hepatosplenomegaly, with the liver measuring 248 mm craniocaudally and the spleen measuring 186 mm in its long axis. According to the HLH-2004 criteria established by the Histiocyte Society, splenomegaly—clinically defined as palpable ≥2 cm below the left costal margin and radiologically as a CT spleen length >12 cm—is one of eight diagnostic features, of which at least five must be fulfilled for HLH diagnosis [6]. This imaging finding, together with extreme hyperferritinemia (16,375 μg/L) and hypertriglyceridemia (1114 mg/dL) on 11–12 December 2024, fulfilled the hepatosplenomegaly criterion in both the HLH-2004 diagnostic criteria [6] and the revised HLH-2024 guidelines [16].

Upon transfer to our tertiary center on 11 December 2024, she appeared markedly more fragile and lethargic, with ECOG performance status declining to 3, accompanied by dyspnea on minimal exertion, profound asthenia, and symptomatic anemia. Laboratory evaluation showed severe pancytopenia (hemoglobin 5.3 g/dL, platelets 86 K/μL, absolute neutrophil count 1.01 K/μL), worsening hyperbilirubinemia (total bilirubin 15.1 mg/dL), elevated lactate dehydrogenase (362 U/L), and extreme hypertriglyceridemia (1114 mg/dL). Reevaluation uncovered hepatosplenomegaly (Figure 1) and hyperferritinemia that had escalated to 16,375 μg/L by December 12. sIL-2R levels measured on December 13 surged to 10,765 pg/mL (normal: 175–858 pg/mL), fulfilling six HLH-2004 criteria: fever (≥38.5 °C), splenomegaly, cytopenias (hemoglobin <9 g/dL, platelets <100 K/μL), hyperferritinemia [>500 (ng/mL]), hypertriglyceridemia (>250 mg/dL), and elevated sIL-2R. Bone marrow biopsy excluded lymphoma, and bone marrow cultures for bacteria, virus, fungus, and acid-fast bacillus (AFB) stain were negative.

**Figure 2:**
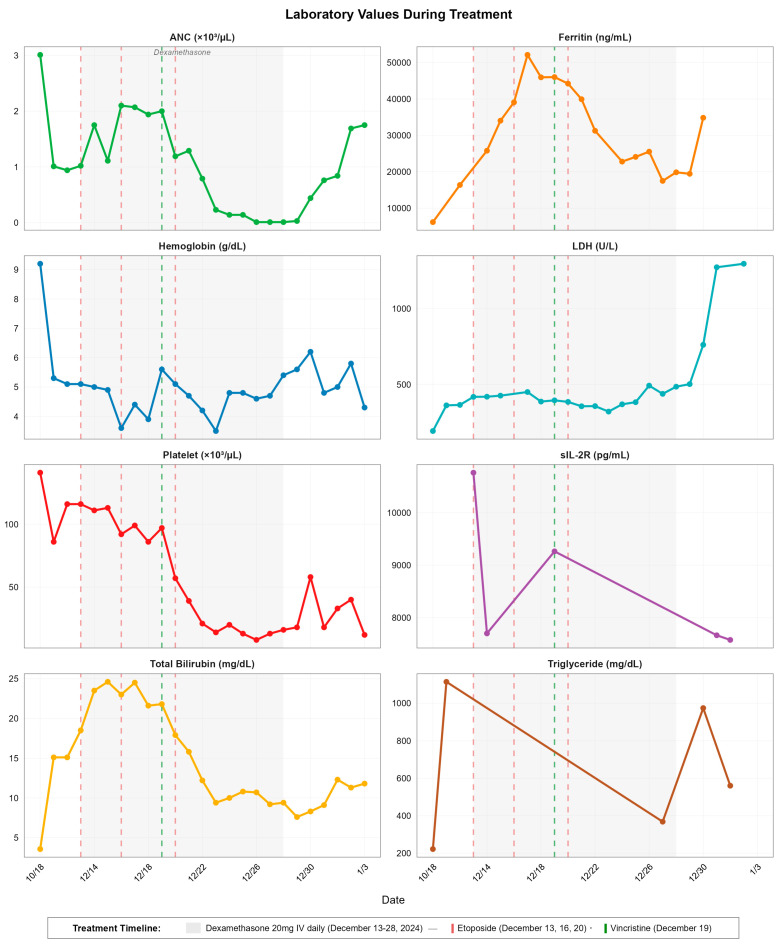
Temporal dynamics of laboratory parameters during hemophagocytic lymphohistiocytosis treatment. Eight-panel time series showing ANC, ferritin, hemoglobin, LDH, platelet count, sIL-2R, total bilirubin, and triglyceride levels from baseline (18 October 2024) through disease progression and treatment. Gray shaded area indicates dexamethasone administration period (13–28 December 2024). Vertical dashed lines indicate etoposide doses (red: 13, 16, 20 December 2024) and vincristine administration (green: 19 December 2024).

Treatment was initiated on 13 December 2024 with dexamethasone (20 mg IV daily) and reduced-dose etoposide (37.5 mg/m^2^ adjusted for hyperbilirubinemia), as per HLH-94 protocol. Laboratory trends during the first week of treatment showed initial worsening: ferritin peaked at 52,129 (ng/mL) on 17 December 2024, while total bilirubin reached 24.6 mg/dL on 15 December. Although sIL-2R declined to 9,264 pg/mL by 19 December 2024, pancytopenia progressively worsened, with platelet counts falling from 116 K/μL at treatment initiation to a nadir of 8 K/μL by 26 December 2024. The absolute neutrophil count demonstrated catastrophic decline from 1.02 K/μL to 0.01 K/μL during the same period, necessitating daily transfusions and granulocyte colony-stimulating factor (G-CSF) support. Details of laboratory response trends to treatment can be found in Table 2 and Figure 2.

Given insufficient response after the second etoposide dose (16 December 2024), vincristine 1.4 mg was added on 19 December 2024 as salvage therapy. The third etoposide dose was administered on 20 December 2024, but further chemotherapy was deferred due to life-threatening neutropenia and infection risk. During this treatment period, she developed bilateral upper extremity DVTs despite severe thrombocytopenia (platelets 57 K/μL on December 20), reflecting HLH’s prothrombotic milieu. Concurrently, CMV viremia exploded from low-level detection to 41,200 IU/mL by 23 December 2024, while EBV DNA persisted with slight interval decrease to 1850 IU/mL.

On December 21, she developed ARDS with bilateral ground-glass and consolidative opacities on CT pulmonary angiography, requiring high-flow nasal cannula (60% fraction of inspired oxygen [FiO2]) and intensive care unit (ICU) transfer. The radiographic findings, combined with her massive CMV viremia and positive parainfluenza, suggested CMV/parainfluenza pneumonia, though HLH-induced ARDS and etoposide pulmonary toxicity remained in the differential. Foscarnet therapy was initiated but failed to halt respiratory deterioration. By 26 December, with ANC at 0.01 K/μL and platelets at 8 K/μL, she required mechanical ventilation for progressive hypoxemic respiratory failure.

Laboratory parameters by the end of dexamethasone therapy (28 December 2024) showed mixed responses: while ferritin decreased to 19,859 μg/L and bilirubin improved to 9.4 mg/dL, critical cytopenias persisted (platelets 16 K/μL, ANC 0.01 K/μL), suggestive of mixed bone marrow suppression from HLH itself and HIV, CMV and EBV viremia. Triglyceride levels also demonstrated late resurgence, from 368 mg/dL (27 December 2024) to 974 mg/dL (30 December 2024). LDH increased from 385 U/L (20 December 2024) to 761 U/L (30 December 2024) and peaked at 1272 U/L (31 December 2024), while Troponin levels began rising on 20 December (24 ng/L 2024) from demand ischemia and surged to 3414 ng/L by 1 January 2025, culminating in ST-segment elevation myocardial infarction on 2 January 202 (troponin peak 4254 ng/L).

Laboratory values in early January showed persistent sIL-2R elevation (7575 pg/mL on January 1), signaling refractory disease despite treatment. The combination of refractory HLH, severe neutropenia precluding further chemotherapy, CMV pneumonia with ARDS requiring mechanical ventilation, and STEMI led to refractory multiorgan failure. Despite partial biochemical improvement, escalating respiratory failure and cytopenias led to ECOG 4 after intubation. Goals-of-care discussions were held, and care transitioned to comfort-focused measures in accordance with the patient’s preferences. She died 21 days post-HLH diagnosis, highlighting the devastating mortality of virus-triggered HLH in profoundly immunocompromised patients.

## 4. Discussion

This case underscores the deadly confluence of advanced AIDS, profound immune dysregulation, and autoimmune hemolysis. The patient’s initial presentation with warm AIHA and transient cold agglutinins masked the underlying HLH, delaying diagnosis until markedly elevated soluble IL-2 receptor (sIL-2R > 10,000 pg/mL) and hyperferritinemia (16,375 μg/L) prompted reevaluation. Such diagnostic ambiguity is common in HIV-associated HLH-AIHA overlap, where cytopenias and hyperferritinemia—hallmarks of both conditions—divert attention from the broader cytokine storm [17]. Notably, the absence of bone marrow hemophagocytosis in this case highlights the limitations of relying on histopathology alone [5], emphasizing the need for early cytokine profiling (e.g., sIL-2R, IFN-γ) in immunocompromised hosts with refractory cytopenias [18,19,20,21,22,23].

Interestingly, the patient was found to have reactivation of CMV and EBV on 13 November 2024, yet both viruses were IgM-negative in October 2024 (Table 1). Seroconversion and rising PCR values in mid-November coincided with persistently low reticulocytes, lending credence to the hypothesis that subsequent viral reactivation impaired marrow compensation rather than initiated hemolysis.

Therapeutic management remains fraught with paradoxes. While etoposide, a cornerstone of HLH-2004 protocols [6], transiently suppressed hyperinflammation here, its myelotoxicity exacerbated pancytopenia, precipitating fatal infections and worsening bleeding—a recurrent issue in AIDS patients with baseline bone marrow suppression. Recent studies advocate cytokine-targeted therapies (e.g., anakinra, emapalumab) to circumvent this dilemma. Anakinra, an IL-1 receptor antagonist, achieved remission in 71% of etoposide-refractory HLH cases without worsening cytopenias [24], while emapalumab’s IFN-γ blockade shows promise in dampening hyperinflammation without compromising antiviral immunity [25]. Rituximab and vincristine, administered to target AIHA, did not yield a significant clinical response for either AIHA or HLH [26,27]. Dexamethasone was continued throughout the course, despite the presence of ARDS due to viral pneumonia and sepsis, as it remains a cornerstone of HLH treatment [24].

A striking feature of this case was the development of bilateral upper extremity DVTs and myocardial infarction at her terminal state despite severe thrombocytopenia, underscoring HLH’s prothrombotic milieu [28]. Studies show that bleeding is common in HLH, but venous thromboembolism also occurs in about 11–13% of adults and is associated with worse survival [29,30]. Elevated tissue factor and VEGF, markers of endothelial injury, drive coagulation in HLH independent of platelet counts—a phenomenon necessitating vigilance even in thrombocytopenic patients [31]. This paradox complicates management, as anticoagulation risks hemorrhage while withholding therapy may accelerate thrombotic events [32].

Prior reports of HLH-AIHA overlap, limited to lymphoma patients, describe median survival of 42 days with etoposide [33], far exceeding the 21-day survival here. This disparity reflects the amplified lethality of HLH in AIDS, where pre-existing immunodeficiency accelerates opportunistic infections [34]. The rapid rise in sIL-2R levels, correlating strongly with EBV viral load (r = 0.72) [23], suggests its utility as an early diagnostic marker in cytopenic AIDS patients. Moving forward, biomarker-driven trials evaluating Janus kinase (JAK) inhibitors (ruxolitinib) [27] or IFN-γ antagonists (emapalumab) [35] are critical to reducing reliance on myelotoxic therapies [36,37,38].

## 5. Literature Review

Hemophagocytic lymphohistiocytosis (HLH) can manifest in HIV patients either as an immune reconstitution inflammatory syndrome (IRIS) upon initiating antiretroviral therapy (ART) or as part of the natural progression of untreated or poorly adherent HIV infection. Although opportunistic pathogens (for example, disseminated histoplasmosis, cytomegalovirus, tuberculosis) and hematologic malignancies have all been reported as potential precipitants, a clear instigating event is not identified in many cases [39,40,41]. Severe CD4-cell depletion and dysfunctional natural-killer–cell cytotoxicity probably lower the threshold for runaway macrophage and T-cell activation, making advanced AIDS an intrinsically permissive environment for HLH and explaining its greater severity and poorer outcomes in this population [42,43]

Diagnostic challenges persist in HIV-associated HLH-AIHA overlap, particularly due to the frequent absence of bone marrow hemophagocytosis in up to 40% of cases [44,45,46]. Soluble IL-2 receptor (sIL-2R) and ferritin have emerged as pivotal biomarkers, with sIL-2R demonstrating superior sensitivity (100% at thresholds >2515 U/mL) compared to ferritin’s specificity (96% at >10,000 μg/L) [11,47,48]. Elevated sIL-2R levels correlate strongly with EBV viral load (r = 0.72), underscoring its utility in early HLH recognition even in patients presenting with AIHA-dominant features [8,11,12,13,14,15,44,45,46,47,49]. Cytokine profiling, including IFN-γ and IL-18, alongside CD163+ macrophage staining, enhances diagnostic specificity but remains underutilized in resource-limited settings [11,50,51]. Advanced imaging modalities, such as positron emission tomography/computed tomography (PET/CT), may aid in excluding lymphoma while confirming systemic immune activation through diffuse splenic and bone marrow uptake [11,52].

Therapeutic management of HLH-AIHA overlap in AIDS is fraught with challenges. Etoposide, a mainstay of HLH-2004 protocols, carries significant risks in immunocompromised hosts, with myelotoxicity (89% neutropenia) and infection-related mortality (64%) exacerbated by pre-existing pancytopenia [53,54,55]. Recent studies highlight cytokine-targeted therapies as safer alternatives: anakinra, an IL-1 receptor antagonist, achieved remission in 71% of etoposide-refractory HLH cases without worsening cytopenias [56,57]. It has shown to achieve disease control in patients unresponsive to corticosteroids, IVIG, ciclosporin, and etoposide. In a separate retrospective cohort of 21 HLH patients, 19 (90.5%) responded to anakinra, with fever abating a median of one day after treatment initiation [56]. Another promising regimen, emapalumab, an anti-IFN-γ monoclonal antibody, binds free and receptor-bound interferon-γ, thereby inhibiting the transduction of interferon-γ signaling, improved 5-year survival rates to 63% in pediatric cohorts [35,58]. It has been approved for adult and pediatric patients with primary HLH who have refractory, recurrent, or progressive disease, as well as for those intolerant of conventional therapies [59]. Emapalumab’s success in neutralizing IFN-γ has validated cytokine-directed therapy in HLH. Similarly, Ruxolitinib, a JAK1/2 inhibitor, normalized ferritin and sIL-2R levels within 7 days in recent AIDS-HLH case reports, offering a promising balance between immunosuppression and infection control [6,60,61]. It is now used as a salvage therapy for relapsed or refractory HLH and may also improve eligibility for allogeneic hematopoietic stem cell transplantation [62]. Additionally, rituximab, a chimeric anti-CD20 monoclonal antibody, has been used to target EBV-driven HLH. In a cohort of 39 patients, rituximab rapidly ameliorated both clinical symptoms and laboratory abnormalities, with a median time to improvement of 14 days [63]. However, its efficacy appears limited in the context of AIDS, likely due to CD20 downregulation and impaired NK-cell function in these patients, underscoring the necessity for more precise cytokine and signaling-pathway–targeted therapies [64,65,66].

Thrombotic complications in HLH, including deep vein thrombosis and myocardial infarction, arise from endothelial injury mediated by tissue factor and vascular endothelial growth factor (VEGF) upregulation, alongside neutrophil extracellular trap (NET)-driven microthrombosis [67,68,69]. Elevated IL-6 and IL-10 directly activate coagulation pathways, creating a paradoxical prothrombotic state despite severe thrombocytopenia [67,68]. This phenomenon complicates management, as anticoagulation risks hemorrhage while therapeutic inertia may accelerate thrombotic events [29,70]. Unfortunately, there are no standardized anticoagulation protocols for HLH. Many clinicians extrapolate from the International Society on Thrombosis and Haemostasis (ISTH) Scientific and Standardization Committee’s framework’s recommendations for cancer-associated thrombosis with thrombocytopenia, i.e., initiating full-dose low-molecular-weight heparin once platelet counts exceed 50 × 10^3^/µL, with close activated partial thromboplastin time (aPTT) and anti–factor Xa (anti-Xa) monitoring and proactive transfusion support. For platelets between 25–50 × 10^3^/µL, intermediate-intensity regimens or mechanical prophylaxis can serve as a bridge until marrow recovery. In rare cases of absolute anticoagulation contraindication, placement of an inferior vena cava filter may be considered on an individual basis [71].

Survival disparities between infection-driven and lymphoma-associated HLH-AIHA in HIV are stark. Lymphoma-associated cases exhibit a median survival of 1.4 months versus 40.9% 1-year survival in infection-driven cohorts [72,73,74]. Early antiretroviral therapy (ART) initiation reduces EBV viral loads by 1–2 logs within 8 weeks, potentially mitigating hyperinflammation and autoantibody production [29,75,76,77,78]. These findings emphasize the critical role of viral suppression in preventing immune collapse and improving outcomes in this high-risk population.

The primary limitation of our case report is its basis on a single patient, which precludes drawing definitive conclusions. Additionally, the existing literature is limited by small sample sizes, retrospective and single-center study designs, diagnostic challenges due to overlapping clinical features with other AIDS-related conditions, variability in diagnostic criteria, and restricted generalizability of findings. Early recognition through sensitive biomarkers including sIL-2R elevation is critical for unmasking HLH in AIHA-dominant presentations. Future studies should explore cytokine-targeted therapies (e.g., anakinra, emapalumab) to reduce reliance on myelotoxic agents like etoposide in immunocompromised hosts. Early palliative care integration is essential given the high mortality of HLH-AIHA overlap in AIDS.

## 6. Conclusions

This report underscores that the hyper-inflammation in HIV-associated HLH-AIHA is multifactorial, arising from a complex interplay of endogenous and exogenous factors that disrupt immune regulation. HIV-induced immunosuppression impairs cytotoxic T lymphocyte and NK cell function, creating a permissive environment for unchecked immune activation. This dysregulation is further exacerbated by infectious triggers (e.g., opportunistic infections) and autoimmune phenomena (e.g., AIHA), which collectively drive a hyperinflammatory state characterized by cytokine storm and tissue injury.

## Figures and Tables

**Table 1:** Summary of EBV and CMV serological tests.

Date	EBV VCA IgM (U/mL)	EBV VCA IgG (U/mL)	EBV EA IgG (U/mL)	EBNA IgG (U/mL)	EBV DNA (IU mL^−1^)	CMV IgM (AU/mL)	CMV PCR (IU mL^−1^)	CMV IgG Ab (U/mL)
13 October 2024	Negative	>750 (positive)	45.8 (positive)	Negative	-	Negative	Negative	2.24 (Positive)
13 November 2024	-	-	-	-	3,310	44.3 (poositive)	Detectable (<34.5)	>10 (positive)
23 November 2024	>160 (positive)	>750 (positive)	48.4 (positive)	>600 (positive)	-	-	-	-
23 December 2024	-	-	-	-	1,850	-	41,200	-

-: Not Available.

**Table 2:** Sequential laboratory values and treatment timeline in virus-associated hemophagocytic lymphohistiocytosis. Values outside normal ranges are highlighted. Baseline values from 18 October 2024 show initial presentation, with subsequent deterioration leading to tertiary center transfer on 11 December 2024. Treatment interventions and corresponding laboratory responses are shown through critical events, including treatment initiation (13 December 2024), nadir values (26 December 2024), and terminal complications (3 January 2025). ANC: absolute neutrophil count; Dex: dexamethasone.

Date	Treatment	Blood Counts			Biochemistry				
		Platelet	Hemoglobin	ANC	Ferritin	sIL-2R	Bilirubin	LDH	Triglycerides
		(×10^3^/µL)	(g/dL)	(×10^3^/µL)	(ng/mL)	(pg/mL)	(mg/dL)	(U/L)	(mg/dL)
Normal Range		150–450	12.0–16.0	1.5–8.0	30–400	175–858	0.1–1.2	140–280	<200
18 October 2024	Baseline	141	9.2	3.01	61.73	—	3.57	193	222
11 December 2024	Pre-treatment	86	5.3	1.01	16,375	—	15.1	362	1114
13 December 2024	Dexamethasone Start; Etoposide #1	116	5.1	1.02	—	10,765	18.5	418	—
15 December 2024	Dex ongoing	113	4.9	1.11	34,046	—	24.6	426	—
16 December 2024	Etoposide #2	92	3.6	2.1	39,099	—	23	—	—
17 December 2024	Dex ongoing	99	4.4	2.07	52,129	—	24.5	450	—
19 December 2024	Vincristine	97	5.6	2	46,018	9264	21.8	395	—
20 December 2024	Etoposide #3	57	5.1	1.19	44,226	—	17.9	385	—
26 December 2024	Dex ongoing	8	4.6	0.01	25,529	—	10.7	492	368
28 December 2024	Dexamethasone End	16	5.4	0.01	19,859	—	9.4	485	—
31 December 2024	-	18	4.8	0.76	—	7663	9.1	1272	974
3 January 2025	STEMI	12	4.3	1.75	—	—	11.8	—	—

## Data Availability

The datasets used and/or analyzed during the current study are available from the corresponding author on reasonable request.

## References

[1] Berentsen S., Barcellini W. (2021). Autoimmune Hemolytic Anemias. N. Engl. J. Med..

[2] Lebrun D., Hentzien M., Cuzin L., Rey D., Joly V., Cotte L., Allavena C., Dellamonica P., Servettaz A., Bani-Sadr F. (2017). Epidemiology of autoimmune and inflammatory diseases in a French nationwide HIV cohort. Aids.

[3] Yen Y.F., Lan Y.C., Huang C.T., Jen I.A., Chen M., Lee C.Y., Chuang P.H., Lee Y., Morisky D.E., Chen Y.A. (2017). Human Immunodeficiency Virus Infection Increases the Risk of Incident Autoimmune Hemolytic Anemia: A Population-Based Cohort Study in Taiwan. J. Infect. Dis..

[4] Fattizzo B., Ferraresi M., Giannotta J., Barcellini W. (2021). Secondary Hemophagocytic Lymphohistiocytosis and Autoimmune Cytopenias: Case Description and Review of the Literature. J. Clin. Med..

[5] Toumeh N., Abu-Zeinah K.F., Godby R.C. (2025). Hemophagocytic lymphohistiocytosis (HLH): A narrative review of the pathogenesis, clinical presentation, diagnosis, treatment, and prognosis. Ann. Blood.

[6] Henter J.I., Horne A., Aricó M., Egeler R.M., Filipovich A.H., Imashuku S., Ladisch S., McClain K., Webb D., Winiarski J. (2007). HLH-2004: Diagnostic and therapeutic guidelines for hemophagocytic lymphohistiocytosis. Pediatr. Blood Cancer.

[7] Hoover J. (2025). Hemophagocytic Lymphohistiocytosis. N. Engl. J. Med..

[8] Brisse E., Wouters C.H., Andrei G., Matthys P. (2017). How Viruses Contribute to the Pathogenesis of Hemophagocytic Lymphohistiocytosis. Front. Immunol..

[9] Behrens E.M., Canna S.W., Slade K., Rao S., Kreiger P.A., Paessler M., Kambayashi T., Koretzky G.A. (2011). Repeated TLR9 stimulation results in macrophage activation syndrome-like disease in mice. J. Clin. Invest..

[10] Thoden J., Rieg S., Venhoff N., Wennekes V., Schmitt-Graeff A., Wagner D., Kern W.V. (2012). Fatal hemophagocytic syndrome in a patient with a previously well-controlled asymptomatic HIV infection after EBV reactivation. J. Infect..

[11] Elliott R.P., Freeman B.P., Meier J.L., El-Herte R. (2022). Acute Cytomegalovirus Illness in an Immunocompetent Adult Causing Intr avascular Hemolysis and Suspected Hemophagocytic Lymphohistiocytosis. Case Rep. Infect. Dis..

[12] Fajgenbaum D.C., June C.H. (2020). Cytokine Storm. N. Engl. J. Med..

[13] Piriou E.R., van Dort K., Nanlohy N.M., van Oers M.H., Miedema F., van Baarle D. (2005). Novel method for detection of virus-specific CD41^+^ T cells indicates a decreased EBV-specific CD4^+^ T cell response in untreated HIV-infected subjects. Eur. J. Immunol..

[14] Legoff J., Amiel C., Calisonni O., Fromentin D., Rajoely B., Abuaf N., Tartour E., Rozenbaum W., Bélec L., Nicolas J.-C. (2004). Early Impairment of CD8^+^ T Cells Immune Response Against Epstein–Barr Virus (EBV) Antigens Associated with High Level of Circulating Mononuclear EBV DNA Load in HIV Infection. J. Clin. Immunol..

[15] Kostense S., Otto S.A., Knol G.J., Manting E.H., Nanlohy N.M., Jansen C., Lange J.M.A., Oers M.H.J.V., Miedema F., Baarle D.V. (2002). Functional restoration of human immunodeficiency virus and Epstein-Barr virus-specific CD8^+^ T cells during highly active antiretroviral therapy is associated with an increase in CD4^+^ T cells. Eur. J. Immunol..

[16] La Rosée P., La Rosée F. (2024). HLH: Diagnostics revisited and improved. Blood.

[17] Jordan M.B., Allen C.E., Greenberg J., Henry M., Hermiston M.L., Kumar A., Hines M., Eckstein O., Ladisch S., Nichols K.E. (2019). Challenges in the diagnosis of hemophagocytic lymphohistiocytosis: Recommendations from the North American Consortium for Histiocytosis (NAC HO). Pediatr. Blood Cancer.

[18] Debaugnies F., Mahadeb B., Nagant C., Meuleman N., De Bels D., Wolff F., Gottignies P., Salaroli A., Borde P., Voué M. (2021). Biomarkers for Early Diagnosis of Hemophagocytic Lymphohistiocytosis in Critically Ill Patients. J. Clin. Immunol..

[19] Liu M., Brodeur K.E., Bledsoe J.R., Harris C.N., Joerger J., Weng R., Hsu E.E., Lam M.T., Rimland C.A., LeSon C.E. (2025). Features of hyperinflammation link the biology of Epstein-Barr virus infection and cytokine storm syndromes. J. Allergy Clin. Immunol..

[20] Petrara M.R., Cattelan A.M., Zanchetta M., Sasset L., Freguja R., Gianesin K., Cecchetto M.G., Carmona F., De Rossi A. (2012). Epstein-Barr Virus load and immune activation in Human Immunodeficiency Virus type 1-infected patients. J. Clin. Virol..

[21] Zhang L. (2022). A common mechanism links Epstein-Barr virus infections and autoimmune diseases. J. Med. Virol..

[22] Boisseau M., Lambotte O., Galicier L., Lerolle N., Marzac C., Aumont C., Coppo P., Fardet L. (2015). Epstein–Barr virus viral load in human immunodeficiency virus-positive patients with reactive hemophagocytic syndrome. Infect. Dis..

[23] Humblet-Baron S., Franckaert D., Dooley J., Bornschein S., Cauwe B., Schönefeldt S., Bossuyt X., Matthys P., Baron F., Wouters C. (2016). IL-2 consumption by highly activated CD8 T cells induces regulatory T-cell dysfunction in patients with hemophagocytic lymphohistiocytosis. J. Allergy Clin. Immunol..

[24] Lee B.J., Cao Y., Vittayawacharin P., É’Leima G., Rezk S., Reid J., Brem E.A., Ciurea S.O., Kongtim P. (2023). Anakinra versus etoposide-based therapy added to high-dose steroids for the treatment of secondary hemophagocytic lymphohistiocytosis. Eur. J. Haematol..

[25] Giri P.P., Pal P., Ghosh A., Sinha R. (2013). Infection-associated haemophagocytic lymphohistiocytosis: A case series using steroids only protocol for management. Rheumatol. Int..

[26] Chellapandian D., Das R., Zelley K., Wiener S.J., Zhao H., Teachey D.T., Nichols K.E., Group E.H.R.S. (2013). Treatment of Epstein Barr virus-induced haemophagocytic lymphohistiocytosis with rituximab-containing chemo-immunotherapeutic regimens. Br. J. Haematol..

[27] Liu X., Zhu X., Zhou X., Xie Y., Xiang D., Wan Z., Huang Y., Zhu B. (2022). Case report: Ruxolitinib as first-line therapy for secondary hemophagocytic lymphohistiocytosis in patients with AIDS. Front. Immunol..

[28] Bai H., Wang Y., Shen L., Luo Y., Tang G., Wang F., Sun Z., Hou H. (2023). The signature and predictive value of immune parameters in patients with secondary hemophagocytic lymphohistiocytosis. Immunobiology.

[29] Valade S., Joly B.S., Veyradier A., Fadlallah J., Zafrani L., Lemiale V., Launois A., Stepanian A., Galicier L., Fieschi C. (2021). Coagulation disorders in patients with severe hemophagocytic lymphohistiocytosis. PLoS ONE.

[30] Croden J., Bilston L., Taparia M., Grossman J., Sun H.L. (2022). Incidence of bleeding and thromboembolism and impact on overall survival in adult patients with hemophagocytic lymphohistiocytosis: A 20-year provincial retrospective cohort study. J. Thromb. Haemost..

[31] Posas-Mendoza T.F., McLeod C., Davis W., Zakem J., Quinet R. (2020). Etiologies and management of haemophagocytic lymphohistiocytosis: Is it time for an updated protocol and targeted treatments?. Rheumatology.

[32] Merrill S.A., Naik R., Streiff M.B., Shanbhag S., Lanzkron S., Braunstein E.M., Moliterno A.M., Brodsky R.A. (2018). A prospective quality improvement initiative in adult hemophagocytic lymphohistiocytosis to improve testing and a framework to facilitate trigger identification and mitigate hemorrhage from retrospective analysis. Medicine.

[33] Migaud P., Müller M., Arastéh K., Hentrich M., Stocker H. (2022). Hemophagocytic lymphohistiocytosis in HIV-associated lymphoproliferative disorders. Ann. Hematol..

[34] Abdelhay A., Mahmoud A., Mostafa M., Jain T., Elseidy S., Fahmawi S., Alkasem M., Ammari O. (2023). Delay in treatment of adult hemophagocytic lymphohistiocytosis is associated with worse in-hospital outcomes. Ann. Hematol..

[35] Merli P., Algeri M., Gaspari S., Locatelli F. (2020). Novel Therapeutic Approaches to Familial HLH (Emapalumab in FHL). Front. Immunol..

[36] Lao K., Sharma N., Gajra A., Vajpayee N. (2016). Hemophagocytic Lymphohistiocytosis and Bone Marrow Hemophagocytosis: A 5-Year Institutional Experience at a Tertiary Care Hospital. South. Med. J..

[37] Qureshi Z., Altaf F., Jamil A., Siddique R. (2024). Rituximab as a Therapeutic Strategy in Hemophagocytic Lymphohistiocytosis: Efficacy, Outcomes, and Survival—Insights from a Systematic Review. Am. J. Clin. Oncol..

[38] Pei Y., Zhu J., Yao R., Cao L., Wang Z., Liang R., Jia Y., Su Y. (2024). Prognostic factors in patients with secondary hemophagocytic lymphohistiocytosis in a Chinese cohort. Ann. Hematol..

[39] Pérez-Lazo G., Maquera-Afaray J., Mejia C.R., Castillo R. (2017). Disseminated histoplasmosis and HIV infection: Case series in a Peruvian hospital. Rev. Chil. Infectol..

[40] Henderson L.A., Cron R.Q. (2020). Macrophage Activation Syndrome and Secondary Hemophagocytic Lymphohistiocytosis in Childhood Inflammatory Disorders: Diagnosis and Management. Pediatr. Drugs.

[41] Zhang K., Astigarraga I., Bryceson Y., Lehmberg K., Machowicz R., Marsh R., Sieni E., Wang Z., Nichols K.E., Adam M.P., Feldman J., Mirzaa G.M. (2025). Familial Hemophagocytic Lymphohistiocytosis.

[42] Liang H., Liu Y., Guo J., Dou M., Zhang X., Hu L., Chen J. (2023). Progression in immunotherapy for advanced prostate cancer. Front. Oncol..

[43] Liang H., Yang Q., Zhang Y., Sun H., Fu Q., Diao T., Wang J., Huang W., Xu Y., Ge N. (2023). Development and validation of a predictive model for the diagnosis of bladder tumors using narrow band imaging. J. Cancer Res. Clin. Oncol..

[44] Chinnici A., Beneforti L., Pegoraro F., Trambusti I., Tondo A., Favre C., Coniglio M.L., Sieni E. (2023). Approaching hemophagocytic lymphohistiocytosis. Front. Immunol..

[45] Ionescu F., Anusim N., Zimmer M., Jaiyesimi I. (2022). Venous thromboembolism prophylaxis in hospitalized sickle cell disease and sickle cell trait patients. Eur. J. Haematol..

[46] Rukerd M.R.Z., Mirkamali H., Nakhaie M., Alizadeh S.D. (2024). GATA2 deficiency and hemophagocytic lymphohistiocytosis (HLH): A systematic review of reported cases. BMC Infect. Dis..

[47] Hayden A., Lin M., Park S., Pudek M., Schneider M., Jordan M.B., Mattman A., Chen L.Y.C. (2017). Soluble interleukin-2 receptor is a sensitive diagnostic test in adult HLH. Blood Adv..

[48] Allen C.E., Yu X., Kozinetz C.A., McClain K.L. (2007). Highly elevated ferritin levels and the diagnosis of hemophagocytic lymphohistiocytosis. Pediatr. Blood Cancer.

[49] Arcenas R.C., Widen R. (2002). Epstein-Barr virus reactivation after superinfection of the BJAB-B1 and P3HR-1 cell lines with cytomegalovirus. BMC Microbiology.

[50] Rocco J.M., Laidlaw E., Galindo F., Anderson M., Rupert A., Higgins J., Sortino O., Ortega-Villa A.M., Sheikh V., Roby G. (2022). Severe Mycobacterial Immune Reconstitution Inflammatory Syndrome (IRIS) in Advanced Human Immunodeficiency Virus (HIV) Has Features of Hemophagocytic Lymphohistiocytosis and Requires Prolonged Immune Suppression. Clin. Infect. Dis..

[51] Leone F., Cotugno N., Casamento Tumeo C., Zangari P., Palomba P., Adorisio R., De Benedetti F., Bracaglia C., Papoff P., Ajassa C. (2023). Hyperinflammatory syndrome in a paediatric patient with a recent diagnosis of HIV/AIDS infection: Hemophagocytic lymphohistiocytosis or immune reconstitution syndrome?. BMC Infect. Dis..

[52] Knauft J., Schenk T., Ernst T., Schnetzke U., Hochhaus A., La Rosée P., Birndt S. (2024). Lymphoma-associated hemophagocytic lymphohistiocytosis (LA-HLH): A scoping review unveils clinical and diagnostic patterns of a lymphoma subgroup with poor prognosis. Leukemia.

[53] Dupont T., Darmon M., Mariotte E., Lemiale V., Fadlallah J., Mirouse A., Zafrani L., Azoulay E., Valade S. (2022). Etoposide treatment in secondary hemophagocytic syndrome: Impact on healthcare-associated infections and survival. Ann. Intensive Care.

[54] Arca M., Fardet L., Galicier L., Rivière S., Marzac C., Aumont C., Lambotte O., Coppo P. (2014). Prognostic factors of early death in a cohort of 162 adult haemophagocytic syndrome: Impact of triggering disease and early treatment with etoposide. Br. J. Haematol..

[55] Bergsten E., Horne A., Aricó M., Astigarraga I., Egeler R.M., Filipovich A.H., Ishii E., Janka G., Ladisch S., Lehmberg K. (2017). Confirmed efficacy of etoposide and dexamethasone in HLH treatment: Long-term results of the cooperative HLH-2004 study. Blood.

[56] Baverez C., Grall M., Gerfaud-Valentin M., De Gail S., Belot A., Perpoint T., Weber E., Reynaud Q., Sève P., Jamilloux Y. (2022). Anakinra for the Treatment of Hemophagocytic Lymphohistiocytosis: 21 Cases. J. Clin. Med..

[57] Naymagon L. (2022). Anakinra for the treatment of adult secondary HLH: A retrospective experience. Int. J. Hematol..

[58] Locatelli F., Jordan M.B., Allen C., Cesaro S., Rizzari C., Rao A., Degar B., Garrington T.P., Sevilla J., Putti M.-C. (2020). Emapalumab in Children with Primary Hemophagocytic Lymphohistiocytosis. N. Engl. J. Med..

[59] Vallurupalli M., Berliner N. (2019). Emapalumab for the treatment of relapsed/refractory hemophagocytic lymphohistiocytosis. Blood.

[60] Hansen S., Alduaij W., Biggs C.M., Belga S., Luecke K., Merkeley H., Chen L.Y.C. (2021). Ruxolitinib as adjunctive therapy for secondary hemophagocytic lymphohistiocytosis: A case series. Eur. J. Haematol..

[61] Maschalidi S., Sepulveda F.E., Garrigue A., Fischer A., de Saint Basile G. (2016). Therapeutic effect of JAK1/2 blockade on the manifestations of hemophagocytic lymphohistiocytosis in mice. Blood.

[62] Wang J., Wang Y., Wu L., Wang X., Jin Z., Gao Z., Wang Z. (2020). Ruxolitinib for refractory/relapsed hemophagocytic lymphohistiocytosis. Haematologica.

[63] Fehniger T.A., Larson S., Trinkaus K., Siegel M.J., Cashen A.F., Blum K.A., Fenske T.S., Hurd D.D., Goy A., Schneider S.E. (2011). A phase 2 multicenter study of lenalidomide in relapsed or refractory classical Hodgkin lymphoma. Blood.

[64] Lim S.H., Vaughan A.T., Ashton-Key M., Williams E.L., Dixon S.V., Chan H.T.C., Beers S.A., French R.R., Cox K.L., Davies A.J. (2011). Fc gamma receptor IIb on target B cells promotes rituximab internalization and reduces clinical efficacy. Blood.

[65] Beers S.A., French R.R., Chan H.T.C., Lim S.H., Jarrett T.C., Vidal R.M., Wijayaweera S.S., Dixon S.V., Kim H., Cox K.L. (2010). Antigenic modulation limits the efficacy of anti-CD20 antibodies: Implications for antibody selection. Blood.

[66] Taylor R.P., Lindorfer M.A. (2010). Antigenic Modulation and Rituximab Resistance. Semin. Hematol..

[67] Yang S.-L., Xu X.-J., Tang Y.-M., Song H., Xu W.-Q., Zhao F.-Y., Shen D.-Y. (2016). Associations between inflammatory cytokines and organ damage in pediatric patients with hemophagocytic lymphohistiocytosis. Cytokine.

[68] Morimoto A., Nakazawa Y., Ishii E. (2016). Hemophagocytic lymphohistiocytosis: Pathogenesis, diagnosis, and management. Pediatr. Int..

[69] Ding J., Hostallero D.E., El Khili M.R., Fonseca G.J., Milette S., Noorah N., Guay-Belzile M., Spicer J., Daneshtalab N., Sirois M. (2021). A network-informed analysis of SARS-CoV-2 and hemophagocytic lymphohistiocytosis genes’ interactions points to Neutrophil extracellular traps as mediators of thrombosis in COVID-19. PLoS Comput. Biol..

[70] Jesudas R., Takemoto C.M. (2023). Where have all the platelets gone? HIT, DIC, or something else?. Hematology.

[71] Held N., Jung B., Baumann Kreuziger L. (2022). Management of cancer-associated thrombosis with thrombocytopenia: Impact of the ISTH guidance statement. Res. Pract. Thromb. Haemost..

[72] Lee C.Y., Wills B., Vardhana S.A., Moskowitz A.J. (2021). Clinical characteristics and outcomes of adult lymphoma-associated hemophagocytic lymphohistiocytosis (HLH). J. Clin. Oncol..

[73] Al-Samkari H., Berliner N. (2018). Hemophagocytic Lymphohistiocytosis. Annu. Rev. Pathol. Mech. Dis..

[74] Nguyen D., Nacher M., Epelboin L., Melzani A., Demar M., Blanchet D., Blaizot R., Drak Alsibai K., Abboud P., Djossou F. (2020). Hemophagocytic Lymphohistiocytosis During HIV Infection in Cayenne Hospital 2012–2015: First Think Histoplasmosis. Front. Cell. Infect. Microbiol..

[75] Piriou E., Jansen C.A., Dort K.v., De Cuyper I., Nanlohy N.M., Lange J.M.A., van Oers M.H.J., Miedema F., van Baarle D. (2005). Reconstitution of EBV Latent but Not Lytic Antigen-Specific CD4^+^ and C D8^+^ T Cells After HIV Treatment with Highly Active Antiretroviral Therapy. J. Immunol..

[76] Keenan C., Nichols K.E., Albeituni S. (2021). Use of the JAK Inhibitor Ruxolitinib in the Treatment of Hemophagocytic Lymphohistiocytosis. Front. Immunol..

[77] Albeituni S., Verbist K.C., Tedrick P.E., Tillman H., Picarsic J., Bassett R., Nichols K.E. (2019). Mechanisms of action of ruxolitinib in murine models of hemophagocytic lymphohistiocytosis. Blood.

[78] Gálvez Acosta S., Javalera Rincón M. (2020). Ruxolitinib as first-line therapy in secondary hemophagocytic lymphohistiocytosis and HIV infection. Int. J. Hematol..

